# Personal recovery for special populations: a qualitative study exploring the role of special interest meetings within 12-step fellowships

**DOI:** 10.1186/s13011-023-00575-6

**Published:** 2024-01-04

**Authors:** Frankco Harris

**Affiliations:** Centre for Criminology, The Faculty of Law, University of Oxford, St Cross Building, St Cross Road, Oxford, UK

**Keywords:** Personal recovery, Special Interest Meetings, 12-Step fellowship, Special Populations, CHIME-D

## Abstract

**Background:**

This study explores how Special Interest Meetings (SIMs), also called topic-specific meetings (e.g., meetings for young people), support recovery in 12-Step fellowships for Special Populations like young people, women and LGBTQIA+ members. Despite their emergence to address the needs of these groups, the specific ways Special Interest Meetings contribute to recovery experiences are understudied.

**Methods:**

In-depth interviews were conducted with 12 participants who had attended Special Interest Meetings in 12-Step fellowships to explore the role of these meetings in recovery. The interviews were analysed using the CHIME-D personal recovery framework (Connectedness, Hope, Identity, Meaning in life, Empowerment, Difficulties).

**Results:**

Special Interest Meetings serve as recovery pathways for Special Populations, incorporating CHIME-D elements to aid recovery and address challenges. This study found four "Special Population Pathways” for recovery: Traditional, Hybrid, SIM-Only, and Outside-Sim Hybrid Pathway.

**Conclusions:**

Special Interest Meetings address specific challenges like discrimination and exclusion faced by Special Populations in recovery. These meetings offer tailored support, deeper connections, improved recovery outcomes, and a sense of empowerment. The existence of "Special Population Pathways” emphasises the ongoing need to address diverse individuals’ specific needs throughout the recovery process.

## Introduction

Recovery is a ‘voluntarily maintained lifestyle characterised by sobriety, personal health and citizenship’ [1]. The concept is also understood as an ongoing multidimensional idiosyncratic process developed and sustained over time [2]. Notably, recovery has gained significant prominence in drug and alcohol policies in the UK, US, Australia and Western Europe [3]. There have also been ongoing calls within the professional treatment field to shift from a short-term, acute care approach to addiction treatment toward a more comprehensive and long-lasting recovery management model [4].

Pathways to recovery include professional clinical treatment and non-professional support like 12-Step mutual-help groups (MHGs) [5]. Consequently, 12-Step fellowships characterised as cost-free and peer-supported [6, 7]) may increase the benefits of professional treatment for substance misuse disorders [8, 9]. 12-Step fellowships also have a proven efficacy: greater 12-Step involvement is associated with better recovery outcomes [10, 11], reduces substance use, improves psychosocial functioning, and lessens healthcare costs [8, 12].

The 12-Step modality of treatment, pioneered by AA, is based on the “disease model” of addiction and focuses on abstinence and fellowship. AA, founded in 1935, was the first 12-Step fellowship; it introduced a framework of recovery, “12 Steps,” and later developed a set of organising principles - the “12 Traditions” [13]. As AA grew, its members diversified by age, gender, ethnicity, sexual orientation, occupational background, and co-occurring problems [4]. Specific AA practices and the social and spiritual change mechanisms through which AA works are complex and multifaceted. While common therapeutic factors and social support have been identified as essential mediators, the specificity of AA’s effects remains an area of ongoing research and debate [11].

The AA model, was subsequently adapted to various substances with the creation of Narcotics Anonymous (NA) in 1953. Over time, it has further expanded to encompass a wide range of addictive, compulsive, and dysfunctional behaviours, leading to the formation of fellowships like Sex and Love Addicts Anonymous (SLAA) in 1976 and Adult Children of Alcoholics (ACA) in 1978. These 12-Step fellowships operate as non-professional, community-based networks. Similarly, Twelve-Step Facilitation (TSF) represents a professional, manualised intervention designed to facilitate individuals’ engagement with 12-Step fellowships like AA [9]. Moreover, there are alternative mutual help groups like Self-Management and Recovery Training (SMART), Double Trouble in Recovery (DTR), and Women for Sobriety (WFS), offering diverse approaches to addiction recovery and support.

Research on 12-Step programs emphasises specific practices such as meeting attendance, sponsorship, spiritual processes, and step-work as “active ingredients” contributing to positive recovery outcomes [14, 15]. Having a “sponsor” – a 12-Step fellow with stable/long-term recovery who offers support and guidance while taking another member through the 12Steps – is a key feature of these programs. The sponsor/sponsee relationship is similar to the professional relationship between a therapist and patient, with the “therapeutic alliance” between individuals seeking support and their sponsors being a strong predictor of improved outcomes in substance use treatment [15]. While regular and consistent meeting attendance over extended periods is the most commonly associated form of 12-Step participation linked to positive recovery outcomes [16–18], service involvement and having a home group are also integral [15]. Consequently, a home group – a specific meeting or group within a fellowship that a member attends regularly and considers their primary or “home” meeting with good cohesion supports recovery success [19].

Special Interest Meetings (SIMs) [20] are essential within 12-Step fellowships, providing support to special populations (SPs) during the recovery process [9]. However, research on the phenomenological experiences of people who attend SIMs in 12-Step recovery is scarce. Although personal recovery has been extensively studied across various domains, a significant research gap exists regarding explicitly exploring the role of SIMs in the recovery journey, impeding a more in-depth exploration of how different meeting types influence individuals’ involvement in fellowship, their ability to stay connected, and the benefits they derive from it [21]. Understanding the influence of pro-social group dynamics, positive identity changes, and meaningful engagement in activities on recovery outcomes is crucial [22]. Also, investigating how factors like race, age, gender, and sexuality impact recovery experiences is important for a broader understanding of recovery [23]. Examining the role of SIMs in addressing these issues is a necessary step in research.

SIMs cater to diverse populations, offering tailored support based on factors like age, gender, sexual orientation, language, and co-occurring problems [24]. Certain SPs, including people of colour, women, and LGBTQIA+ individuals, face heightened risks, disparities in healthcare, and challenges related to acceptance and well-being [25, 26]. Consequently, Kelly and Yeterian argue that questions arise about whether mutual help groups like 12-step meetings are less suitable for SPs [21]. While traditional meetings have shown benefits for SPs, attending SIMs tailored to their specific needs may enhance their recovery experiences.

Feeling uncomfortable or lacking support within a group, resulting from being part of a specific population, has been recognised as a significant obstacle to participating and actively engaging in 12-Step recovery [23], particularly for SPs who experience chronic exclusion or trauma due to stigma [27]. Similarly, research has shown that individuals with social anxiety may have difficulty participating in group settings, which is common in addiction treatment programs and 12-step fellowships. This anxiety affects fundamental aspects of recovery such as 12-Step meeting attendance, engaging with a therapist, speaking in group therapy, and seeking a sponsor [28].

Safety is a critical aspect of 12-Step fellowships, and feeling unsafe within these groups can impede personal recovery. For instance, to keep newcomers safe, Narcotics Anonymous suggests that members work with those of the same sex to mitigate the risk of sexually predatory or inappropriate behaviour [29]. Issues related to safety in fellowships also include violence, bullying, sexual harassment, financial coercion, racial discrimination, sexual orientation or gender intolerance, and pressure to adopt specific medical treatments or medication viewpoints [30]. Consequently, by creating safe and supportive environments, SIMs can be crucial in supporting individuals in recovery from SPs.

Peers play a crucial role in recovery as they form positive social networks contributing to social recovery capital [31]. However, a peer in 12-Step research is not adequately defined. According to Penney [32], peers share demographic or social similarities, and support involves deep empathy, encouragement, and assistance within a reciprocal relationship. In the context of 12-Step fellowships, individuals construct their identities based on various characteristics, some of which may conflict. Sharing a common addiction problem does not automatically make individuals peers, as their ability to support each other effectively may vary, highlighting the importance of personal and relational peer support. For example, LGBTQIA+ individuals in recovery may prefer to associate with LGBTQIA+ peers only or choose a sponsor who shares this identity.

CHIME is a conceptual framework originally developed for personal recovery in mental health, which includes five recovery processes that support personal recovery: Connectedness (e.g., peer support, belonging, relationships and community); Hope and Optimism about the future (e.g., hopes, dreams, positive motivation, and aspirations); Identity (developing a new positive identity, exploring dimensions of identity, and overcoming stigma); Meaning in Life (spirituality and meaningfulness from roles, goals and experiences in life); and Empowerment (focusing on personal strength and control over life) [33]. CHIME has proven effective in researching substance use disorders and mutual help groups [2, 21, 34, 35].

Stuart et al. extended CHIME to form the CHIME-D framework, introducing “D” for Difficulties, which underscores challenges in recovery [36]. Their argument highlights the need to address varying recovery experiences and recognise individuals’ struggles and barriers during recovery. Similarly, Laudet suggests that CHIME can sometimes overly emphasise positivity, potentially diminishing individual empowerment by blaming individuals and imposing a professional perspective. It’s imperative to grasp diverse recovery experiences and the associated struggles to truly understand the recovery process [37]. For instance, SPs in recovery encounter significant challenges such as severe poverty, frequent exposure to violence and victimisation, and intense social exclusion [38].

By taking a broader perspective that encompasses safety, recovery capital, peer support, and the CHIME-D framework, researchers can comprehensively understand the effectiveness, challenges, and potential barriers within the 12-Step recovery landscape. This study seeks to expand our knowledge of the recovery process in 12-Step fellowships by explicitly examining the role of SIMs and exploring the experiences of SPs. This will contribute to promoting inclusive and tailored support and improving treatment outcomes for diverse populations within the recovery landscape and address the gap in research in this field.

## Methods

### Research design

In this phenomenological study, I employed qualitative research methods in the form of in-depth interviews to delve into the personal recovery experiences of 12-Step members from SPs who have attended SIMs. Guided by the research question, “What role do Special Interest Meetings play in 12-Step recovery?” the study aimed to comprehend recovery holistically by grasping the lived experience of individuals from SPs in 12-Step recovery. Understanding the lived experiences of individuals undergoing this process is crucial to gaining a comprehensive understanding of recovery [39]. Using the CHIME-D framework, an inductive reasoning approach was employed to analyse the recovery-supportive elements of SIMs. The CHIME-D master themes, pre-identified, have proven valuable for comprehending personal recovery. Therefore, this framework was chosen to analyse the study’s context-specific themes, allowing for the exploration of subthemes.

### Participants

A nonprobability sampling approach was employed for the recruitment of 12 participants, which included convenience (*n* = 7), purposive (*n* = 4), and snowball sampling (*n* = 1) methods. The decision regarding the sample size was made with the goal of keeping the study manageable within the project’s boundaries. Additionally, I took into account the success of related qualitative research that utilised a similar sample size, as demonstrated in Dekkers’ study from 2020 [35]. As a member of various 12-Step fellowships, I utilised my network to identify and recruit participants by disseminating a research poster within the 12-Step community. I initiated direct communication with particular individuals whom I believed embodied diverse characteristics. Although efforts were made to enlist international participants, all participants turned out to be citizens of the United Kingdom. Eligibility criteria included: (1) being 18 or older; (2) having at least 90 days of 12-Step fellowship membership; (3) having engaged in the 12-Step process, and (4) having a sponsor. Combining these criteria, the study aimed to include participants actively involved and relatively stable in their 12-Step recovery journey.

### Instruments and data collection

Ethical approval for this study was obtained from The London School of Economics Research Ethics Committee, and all participants provided informed consent. To uphold participant confidentiality and anonymity, all real names were substituted with pseudonyms, such as “Participant 1”. 12 in-depth interviews were conducted with 12Step members from SPs via Zoom in May 2022 to explore their personal recovery experiences. To initiate discussions, I asked participants to self-define their gender, sexuality and race, as allowing them to self-identify promotes inclusivity, improves data accuracy, and contributes to more informed and equitable research outcomes. Selfidentification respects individuals’ rights to define and express their own identities. It acknowledges that individuals are the experts on their own experiences and allows them to represent themselves authentically. This approach also recognises the complexity and diversity within these categories, capturing the nuances that standardised categories might overlook, which is vital concerning SPs.

Participants were then asked to share their “Experience, Strength, and Hope” for 10 minutes [40], allowing them to discuss their unique recovery journey in a familiar format - similar to 12-Step meetings - which offered them the chance to share their experiences without being constrained by predefined research questions. Based on my familiarity with 12-Step meetings, I anticipated that Experience, Strength and Hope messages often align with the themes of CHIME-D. During this segment, I took notes of their perceived difficulties, emotions, or feelings, which aided in exploring the participant’s recovery process more deeply in the remainder of the interviews with semi-structured interview questions Table 1.

**Table 1:** Participant characteristics

Participant	Age	Race	Gender	Sexuality	12-Step Fellowship Attended	Time in Recovery	Special Interest Meeting (SIM) attended	Pathway used in Recovery
1	39	White	Male	Heterosexual	SLAA,NA AA,ACA	4 years	Men	Traditional
2	33	Displaced African	She/Her/They/Them	Queer lesbian	NA	5 years	Women, POC, LGBTQIA+, “Black Recovery Matters”	Outside-SIM Hybrid
3	49	Brisitish Pakistani	Male	Homosexual	NA,CMA AA,CA	8 years	LGBTQIA+, POC, Men	Hybrid
4	35	Mixed Carribean	Female	Bisexual	NA,CA,AA	7 years	Women,LGBTQIA+	Hybrid
5	43	White Other	Trans Female	Hetero-flexible	NA,AA, SLAA,OA, WA,ITTA	9 years	LGBTQIA+, Women; Trans Sex Workers	Hybrid
6	24	White	Genderqueer	Queer-Pansexual	N,AA	17 months	LGBTQIA+ Young People, Women, Non-binary, LGBTQIA+	Outside-SIM Hybrid
7	63	White	Male	Homosexual	NA,AA	13 years	LGBTQIA+, Times of Illness	SIM-Only
8	40	White European	Non-binary	Pansexual	NA,CMA, ABA,AA	8 years	LGBTQIA+, “Illness in Recovery” Men, Language-Specific	Hybrid
9	57	Afro Carribean-Mixed	Female	Heterosexual	CA,AA,NA	19 years	Women	Hybrid
10	46	Black British; Carribean	Male	Heterosexual	NA,CODA,AA	8 years	“Black Recovery Matters”	Hybrid
11	50	Mixed	Male	Homosexual	CMA,NA,AA	5 years	LGBTQIA+	Traditional
12	33	Black	He/They	Queer	ACA,CODA,NA, AA,SLAA	4 years	POC,BIPOC, LGBTQIA+	Hybrid

Abbreviations defined: *AA* Alcoholics Anonymous, *ABA* Anorexics and Bulimics Anonymous, *ACA* Adult Children of Alcoholics and Dysfunctional Families, *BAME* Black, Asian and Minority Ethnic, *BIPOC* Black, Indigenous, and Other People of Colour, *CA* Cocaine Anonymous, *CMA* Crystal Meth Anonymous, *CODA* Co-Dependents Anonymous, *ITTA* Internet and Technology Addicts Anonymous, *LGBTQIA+* Lesbian, Gay, Bisexual, Transgender, Queer/Questioning, Intersex and Asexual, *NA* Narcotics Anonymous, *SLAA* Sex and Love Addicts Anonymous, *OA* Overeaters Anonymous, *POC* People of Colour, *WA* Workaholics Anonymous

Pathway description:

Traditional - Involves attending meetings where individuals primarily identify as addicts or with a specific issue in a 12-Step fellowship.

Hybrid - Includes participation in both SIMs and traditional meetings to find support and connection in both settings.

SIM-Only - Exclusively entails attending SIMs within a specific fellowship.

Outside-SIM Hybrid - Combines the support of a SIM formed outside a primary fellowship with traditional fellowship meetings.

### Data analysis

The data collected underwent a framework analysis process according to the predefined codes in the CHIME-D framework adopted for this study. This framework analysis process occurred through distinct stages to extract meaningful insights and develop descriptive and explanatory conclusions clustered around emergent themes.

#### Stage 1: data transcription and immersion

The first stage commenced with verbatim transcription of interviews, facilitated by the online transcription service TRINT. Subsequently, these transcriptions underwent a manual review, rectifying errors and allowing for immersion within the dataset. This thorough engagement with the transcriptions fostered an in-depth understanding of the nuanced content.

#### Stage 2: immersed Reading and analytical notes

The subsequent stage involved repeated readings of the transcripts. This iterative process was accompanied by creating analytical notes, contributing to a heightened familiarity with the data and uncovering latent insights within the narratives, including how safety and identity in recovery are multidimensional concepts.

#### Stage 3: applying the CHIME-D framework

The CHIME-D framework’s master themes were then systematically applied, categorising the data segments through colour coding. This technique facilitated the identification of overarching themes that aligned with the CHIME-D components, and concurrently, the subtheme of safety emerged and was pinpointed and tagged within the dataset.

#### Stage 4: data charting and matrix generation

The fourth stage saw data organisation into a matrix, achieved through a spreadsheet. This matrix was structured using colour-coded columns corresponding to the CHIMED framework’s components from the previous step. The matrix rows were divided into sections dedicated to individual participants, thus representing how their experiences correlated with the framework and the additional theme of safety.

#### Stage 5: interpretation and synthesis

Over several weeks, the data interpretation process unfolded, delving deeper into the data’s intricate connections, narrative threads, disparities, and emergent subthemes. This interpretative phase led to a deeper understanding of underlying patterns and relationships, culminating in comprehensive insights. Connectedness, Hope, and Identity were featured significantly in SIMs, whilst Meaning In Life featured less prominently. The data strongly substantiated the category of Difficulties. As such, it is presented separately at the beginning of the analysis, which follows, as the challenges experienced by SPs formed the basis for SIMs.

## Results

### Connectedness

Connectedness includes peer support; support groups; relationships; being a part of a community [33].

SIMs were used to build strong connections and support networks within 12-Step recovery communities:

*‘Special interest groups have helped me dramatically to find my tribe. I [discovered] my sponsors through these groups [and] another level of recovery that feels almost tailored to my needs.’*(Participant 5).

*‘[In SIMs there are] fellows that travel the same journey. Fellows that you have more in common with than just the meetings. Fellows that you [can] bond with outside of the meetings’*(Participant 11).

Participants spoke about the value of connecting with like-minded peers for a deeper level of support in SIMs:

*‘I understand when we share our struggles. When another woman shares her struggles, it allows me to share mine, and we’ve got that compassion and understanding.’*(Participant 9).

*‘It’s really nice to be at a meeting where [people] have similar situations. It’s not the same thing that’s happened [to them], but the same powerlessness, feelings of shame and whatever related.’*(Participant 7).

SIMs provided an environment where individuals could connect and delve into various dimensions of their own identities:

*‘Personal recovery [in SIMs] means [connecting] with all parts of me – however it chooses to express itself. To be able to sit with it and acknowledge it.’*(Participant 2).

*‘Going to the black meeting allowed me to be like, oh, there’s more to this story that I’ve just packed away or stifled from sharing.’*(Participant 12).

The power of friendships formed in SIMs was a topic among several participants, including how valuable these connections can be:

*‘I’ve got friends I have common interests with, other than using- people I can talk to on a deep level.* (Participant 6).

*‘I guess I created my first real friendship with other gay men and trans women [which I never had].’*(Participant 8).

Conversely, Participant 5 spoke about the importance of connecting with the broader fellowship community beyond SIMs: *‘I don’t want to pigeonhole myself and make my recovery smaller. I want to have a bigger fellowship, a bigger network of people*. *.. because I strongly believe that diversity is our power; is our strength - is my strength.’*

Participants discussed how SIMs allowed them to build strong connections and support networks, often leading to profound personal transformations. These connections extended beyond the typical fellowship meetings, offering a deeper sense of belonging and understanding. This highlights the potential of SIMs to address a critical need within the recovery community.

### Hope and optimism about the future

Hope includes belief in the possibility of recovery; motivation to change; hopeinspiring relationships; positive thinking; valuing success, and having dreams and aspirations [33].

Reflecting on the inspiring connections made with individuals also recovering from co-occurring illnesses, participants found hope and strength in their shared experiences:

*‘[Sharing about my illness] helped me to share what I was going through, and it helped other people because I was getting through it [sober].’* (Participant 5).

*‘I met people not with the same pain, but [hearing other people] who learned how to deal with the mental isolation the pain brings to your life allowed me to do the same.’* (Participant 8).

Several participants reported feeling renewed hope and belief in the possibility of recovery. Joining a community of people who you identify with can be a powerful source of support and motivation:

*‘That[SIM] really gave me a sense of healing, and it was really beautiful to come on the screen and see people who look just like me and listen to their experiences.* (Participant 10).

*‘I didn’t realise how many young people there would be. [That] was really soothing because I didn’t think young people could get [sober].’*(Participant 6).

SIMs were a recovery resource where participants found their home group:

*‘I found a home group where I feel comfortable. .. and a whole new take on my recovery has happened. My hope is always found and renewed in my home-group.’*(Participant 2).

*‘Everyone is non-binary. I’ve had this homegroup group struggle and journey for a long time, but this is the first time I’ve actually felt like I have a home-group.*(Participant 12).

The participants’ experiences in SIMs gave them hope and motivation for recovery. They shared how being part of a community where they felt understood and supported inspired them to believe in the possibility of recovery. SIMs served as a source of renewed hope, especially for those who might have felt isolated or doubtful about their recovery prospects.

### Identity

Identity includes dimensions of identity; rebuilding/redefining a positive sense of identity; and overcoming stigma [33].

Participant 5 discussed the various ways they identify and what that meant for them: *‘These are important identities that I have to live by. .. they don’t necessarily fully define me, but they definitely express a big part of me, so I want to be able to feel free to express those identities in the space that I share with other people and where I get the help that they need for my sobriety, for my recovery.’*

Some participants highlighted the importance of connecting and identifying in various ways with others through SIMs, and how building such relationships can add value to recovery experiences: *‘I’d say that specialist meetings are valid, particularly [those] around race and identification abilities, different disabilities, sexualities, [and] sex work.’*(Participant 2).

*‘I go to mainstream meetings, I go to LGBT meetings, I go to BAME meetings, and I identify in all of those meetings and get advice on different types of things. Whether it’s my ethnicity, being a parent, whether it is identifying with LGBT issues, etc.’* (Participant 3).

Participant 8 spoke about how a sense of identity could be lost if they did not have access to SIMs:*‘[Not having SIMs] would be very difficult because I would lose the sense of identification that I have here.’*

Participant 5 shared an insight about the level of identification that can be found in SIMs: *‘I really enjoyed going to that meeting because the identification I get is much higher than the average meeting that I go to.’*

Some participants found that engaging with SIMs helped them reclaim their sense of self-worth and identity and assisted with personal growth and development:

*‘Going to the specialist meetings has really helped me out...they helped me to grow and blossom, be comfortable, get rid of a lot of my internalised homophobia, and be comfortable with myself.’*(Participant 3).

*‘To see other people from my same background helped me to realise, actually, no, this is about who I am, not where I am from.’*(Participant 8).

Participant 11 spoke about group dynamics in SIMs and how being a part of a SIM does not mean there will be automatic identification with others: *‘I didn’t find any identification [in that SIM]. I didn’t have anything in common with the LGBTQIA+ people in that meeting except that they were LGBTQIA+.’*

Participants emphasised the significance of identity within the context of SIMs. They found value in attending meetings aligned with their specific identities, such as race, gender, or sexuality. These meetings allowed individuals to share their unique experiences and challenges, contributing to a sense of belonging and self-acceptance. SIMs also played a crucial role in helping them reclaim their sense of self-worth and personal growth.

### Meaning in life

Meaning In Life includes meaningful recovery experiences; spirituality; quality of life; meaningful life; social goals/roles; and rebuilding life [33].

Several participants shared their experiences of initially receiving support in a SIM and then being able to give back by being of service in these meetings:

*‘That [SIM] meant a lot to me as a newcomer. [Coming back] is like a full-circle moment. I feel like I’m giving back to the room that really helped me when I was fresh in.’*(Participant 6).

*‘[SIMs] are the contribution to my recovery! And I, through service, [contribute] back because [it] is a pillar of my recovery.’*(Participant 2).

A black female participant who did not see other black people in her first meeting and wanted to stay away from meetings discovered the power of her presence. Instead of not coming back, she realised that her unique perspective and insights could be helpful in traditional meetings: *‘[A guy told me]“Why don’t you stay [in the meeting], so when the next black person walks in, you can be there for him?” That’s why I stayed. I realised that gave me a purpose to go back, [and] that I [could] be of help to someone.’*(Participant 9).

Participants shared how SIMs played a meaningful role in their recovery journey.

They highlighted the importance of giving back to their SIM community and how their experiences in SIMs empowered them to contribute to others’ recovery. This section reflects the idea that SIMs serve as a platform for individuals to find purpose and meaning by supporting similar peers in their recovery journey.

### Empowerment

Empowerment includes personal responsibility; control over life; and focusing on strengths [33].

For seven participants, taking personal responsibility for their recovery was crucial. They assisted in the creation of groups and spaces dedicated to specific issues and SPs:

*‘We started our first Crystal Meth Anonymous meeting. The reason behind that was that we didn’t feel that in the other fellowship, there was a place for us crystal meth addicts to talk openly about our struggles with specific ways of taking drugs.’* (Participant 11).

*‘We all came together and decided to create a space for ourselves so that we can do that freely. And it’s just turned into a really beautiful and loving space.’*(Participant 2).

Several participants helped to create novel SIMs, which combined fellowship programs and created unique structures to better aid individuals in recovery:

*‘[Black Recovery Matters] does follow the structure of [Narcotics Anonymous] NA, but it sort of has its own [structure] because it’s not part of the fellowship. It has its own sort of jurisdiction. It follows its own road, basically.’*(Participant 10).

*‘It’s an online meeting that we started in Berlin. .. and it’s a combination of [fellowships]. So we use literature from [different] fellowships, which can be said to be quite controversial.’*(Participant 5).

Participant 1 shared how SIMs empowered them to transition to traditional meetings. SIMs provided a safe place to stabilise and were used as a transition point: *‘I came out of the special interest meeting maybe two months ago. It felt like I was able to come out and be back involved in mixed meetings.’*

The participants’ accounts highlight that empowerment in SIMs involves personal responsibility, autonomy, and a strengths-based approach. Their experiences underscore the importance of tailored and adaptable support options within recovery, revealing the resilience and flexibility inherent in individuals’ journeys toward sobriety.

### Difficulties

Difficulties include challenges, barriers, and difficulties faced in recovery [33].

A male participant shared his struggles with attending mixed-gender meetings, expressing how challenging it can be to navigate these situations: *‘I did mixed meetings to start with, and I found that [they were] full of really beautiful women. It was pretty overwhelming, and it became unbearable being in [those] meetings. I felt like for my sanity and to get any kind of recovery, I probably needed to do men’s meetings, which I did.’*(Participant 1).

Several participants spoke about the difficulty of dealing with various forms of harmful behaviour in traditional meetings:

*‘I’ve heard black jokes, and I was trying to challenge that [but] was constantly being rejected and gaslit.*. *.I felt crushed. So helpless. So unsafe.’*(Participant 2).

*‘I have heard about how some men can be predatory around women, and actually, I’ve almost experienced that before. .. you know, it’s an issue!. .. There’s a lot of sick people!. .. and a lot of men with sexual issues, and there is predatory behaviour!’(Participant 4).*

As 12-step fellowships strive to be more inclusive, some members feel threatened by the exclusivity of SIMs. Some see SIMs as a barrier to the wider fellowship:

*‘The moment we turn them away and say,” Sorry mate, this is a meeting for black people,” “This is a meeting for Latinos,” “This is a gay or lesbian meeting”... as soon as someone says, “you can’t be here” and sends them away, it ceases to be a meeting.’*(Participant 10).

*‘I feel like [the SIM] became very one dimensional, and I felt like it had become quite cultish and sort of separate. .. and it felt like they were going against some of the Traditions.’*(Participant 1).

Participants experienced challenges and difficulties, including issues related to mixed-gender meetings and harmful behaviour within traditional meetings. The experiences highlighted and illustrate the complex dynamics at play when integrating SIMs within fellowships. While some participants found it challenging to navigate mixedgender meetings and advocated for specialised meetings, concerns were raised about potential divisiveness. The participants’ experiences call for a thoughtful and inclusive approach to addiction recovery that respects individual needs while maintaining the core principles of fellowship and support.

### Safety

Within the exploration of participants’ experiences, the theme of safety emerged prominently during the coding process, encompassing sub-themes such as general safety, physical well-being, perceived safety, and the significance of safe spaces.

Ten participants spoke about the value of SIMs being a place of safety:

*‘It feels really powerful to share this space with people that make it feel safe and welcoming.’*(Participant 5).

*‘[SIMs] help, especially the newcomer, feel safer and be with more like-minded and similar people.”*(Participant 6).

Participant 12 shared their experience about the impact of feeling unsafe during their recovery journey: *‘I don’t feel like it’s safe enough that I can settle.’*

Participant 11 shared a unique perspective, expressing that they did not personally feel the need for safe spaces. This viewpoint opens up a larger conversation on the importance and necessity of safe spaces in various contexts:

*‘Personally, I don’t feel the need for a safe LGBTQIA+ space in recovery. I appreciate and acknowledge the importance of that for others, and I recognise that their experience and my experience in that area is different. For me, I’m in a meeting. I’m here because I want recovery. I suppose I’m seeing a lot of this from the perspective of somebody who is a few years clean, and I have to put myself in the position of a newcomer.’*

Participants discussed the value of SIMs as safe and welcoming spaces. The importance of safety within SIMs highlights the need for spaces where individuals feel secure and supported in their recovery journeys. The diversity of perspectives on the necessity of safe spaces within recovery prompts reflection on creating environments that cater to varying comfort levels and needs while maintaining the overall safety and inclusivity of the recovery community.

## Discussion and analysis

CHIME-D captures the supportive recovery elements of SIMs within 12-Step fellowships. The data shows that the themes of Connectedness, Hope, Identity, and Empowerment feature strongly in SIMs, whilst Meaning In Life is featured less. The theme of Difficulties is presented separately at the beginning of this analysis, as the challenges or barriers experienced by some people from SPs form the basis for SIMs and play a leading role in the research. The additional theme, “Safety”, discovered during the coding process, is also discussed.

### Difficulties

Recovery journeys often present considerable challenges, particularly for individuals belonging to SPs. SIMs have emerged as a valuable solution in response to these difficulties, providing a sanctuary for SPs to address their unique struggles and advance their recovery. The current research corroborates existing studies, confirming that SPs frequently encounter challenges related to acceptance and well-being [25]. The insights gained from participant interviews further highlight the depth of these challenges, with descriptors like “unbearable,”“unsafe,”“rejected,”“gaslit,”“crushed,”“predatory,” and “pain” underscoring their experiences.

For instance, a male participant shared his experience of finding mixed meetings with women overwhelming and unbearable, leading him to seek solace and progress through men’s meetings (Participant 1). This illustrates how the presence of individuals from the opposite sex can hinder the recovery journey for specific SPs. Moreover, participants recounted grappling with distressing behaviours within traditional meetings. One participant (Participant 2) said encountering discriminatory jokes and feeling rejected and gaslit when attempting to address them led to feelings of helplessness and insecurity. Another participant (Participant 4) shed light on the problem of predatory behaviour, indicating that some individuals with sexual issues exhibited such behaviour in the meetings. These accounts underscore the detrimental impact of harmful behaviour on SPs’ recovery experiences and the significance of creating safe spaces like SIMs.

Acknowledging these challenges and nurturing a supportive community environment remain vital for SIMs to effectively address critical needs within 12-Step recovery. While the CHIME-D framework inherently encompasses Difficulties encountered in traditional 12-Step meetings, the current findings accentuate that these challenges assume greater prominence within SIMs due to the specific hurdles SPs face. This divergence from the conclusions drawn in Dekkers et al.’s study [35], which suggested an interconnected relationship between Difficulties and other CHIME categories, underscores my contention that these identified challenges warrant distinct consideration beyond the broader CHIME themes. My research highlights the enduring exclusion that SPs experience, evident in instances of discrimination and judgment within 12-Step fellowships, which substantially impedes their recovery journey and can be trauma-inducing [25]. Furthermore, some members’ resistance to integrating SIMs into 12-Step fellowships leads to cases where SPs’ concerns about racism, sexism, homophobia, or transphobia are invalidated, leading to members from SPs feeling a sense of despondence and disconnection.

Exclusion, insecurity, and difficulties in connecting experienced by SP members in traditional meetings directly influence fundamental program-specific practices such as meeting attendance and fellowship—cornerstones of successful recovery. Conversely, SP members who feel excluded in traditional 12-Step meetings find renewed hope for recovery through participation in SIMs. While SIMs play a pivotal role in establishing connections, it is crucial not to underestimate the importance of forging connections within the broader 12-step fellowship. The exclusivity of SIMs can inadvertently foster division within 12-Step fellowships. This concern is echoed in recent research by Dekkers et al. [35], which highlights the potential risks of disunity in the context of recovery. The robust connections cultivated in recovery-supportive groups like SIMs may unintentionally foster an “us” versus “them” dynamic, erecting barriers that obstruct members from seeking or leveraging support beyond their immediate social circles.

The difficulties explored in this study have far-reaching implications beyond their impact on Special Populations within SIMs. These challenges resonate across the entire spectrum of recovery pathways, including professional treatment modalities, and they underscore the need for a holistic approach to address these issues and cater to the unique needs and vulnerabilities of individuals within these populations. Research has highlighted how clinicians and treatment should be attuned to difficulties faced by individuals when designing interventions, ensuring they are culturally sensitive, inclusive, and trauma-informed [41, 42].

Some SPs (e.g., people of colour, women, LGBTQ+) often face heightened risks to health, disparities in healthcare treatment, and struggle with safety, acceptance, and well-being issues [25]. These disparities and challenges emphasise the importance of addressing inequities in access to specialised treatment options. By incorporating cultural sensitivity, inclusivity, and trauma-informed approaches into interventions, policymakers and healthcare providers could consider these disparities when allocating resources and design healthcare systems to ensure that all individuals, including SPs, have equitable access to specialised treatment.

#### Connectedness

The participants’ experiences within SIMs illustrate the concept of connectedness outlined by Leamy et al. [33]. The formation of peer support, relationships, and a sense of community is evident through their narratives. A central theme was that SIMs help create strong connections and support networks within 12-step recovery communities for individuals with shared experiences. The findings support Dekker’s study, which concluded that the essential factor that seemed to promote recovery in NA was a sense of connection, underscoring the relational aspect of recovery [35].

The connections can lead to deeper bonds, allowing SP members to identify with fellow members who share similar journeys (Participant 11). The value of connecting with like-minded peers is prominent in the participants’ discussions, as they spoke about sharing struggles, common experiences, and feelings of compassion and understanding beyond their primary addiction or issue (Participant 9). SIMs also allow individuals to explore different aspects of their identities, enhancing the quality of connections by enabling them to connect with all parts of themselves (Participant 2).

SIMs act as a conduit for individuals to find their “tribe” and establish relationships beyond mere meeting attendance (Participant 5). The concept of a “tribe” in these meetings can foster a sense of community and deeper relationships among members, but it can also lead to exclusivity, groupthink, and a loss of individuality, as observed by different participants. Fellowships struggle to ensure that the positive aspects of SIMs are harnessed while mitigating the negative aspects associated with exclusivity and conformity, which can lead to SIMs feeling “cultish”, as discussed by Participant 1.

#### Hope and optimism about the future

SIMs foster a belief in the possibility of recovery, the motivation to change, and the fostering of hope-inspiring relationships for those from SPs. The connections formed in SIMs provide strength and hope, demonstrating how shared experiences could inspire recovery efforts (Participant 5). Witnessing others’ journeys of overcoming mental isolation and pain within these specialised communities, kindles hope and motivates SP members (Participant 8). Being part of a community one can identify with becomes a potent source of support and motivation, inspiring individuals to pursue recovery (Participant 10). Participant 6’s realisation that young people could achieve sobriety through the SIM experience illustrates the transformative power of hope.

Some members from SPs choose SIMs as their homegroups, emphasising the significance of these settings as places of hope. Participant 2 shared that their homegroup brought a new perspective to their recovery, consistently renewing their sense of hope. Participant 12 spoke about finding a homegroup where they felt a sense of belonging, highlighting how it positively impacted their recovery journey. These accounts collectively underscore how SIMs serve as catalysts for hope, motivation, and transformative connections, solidifying their vital role in fostering recovery within the context of shared experiences.

#### Identity

The concept of identity within the context of SIMs takes on a multidimensional nature and is featured prominently in this study. SP members construct their identities based on a range of characteristics and contexts. Importantly, this dynamic is underscored by the understanding that primary identification solely as an addict may not suffice for some SP members to recover within the framework of 12-Step fellowships effectively. The data also highlights the concept of “levels of identification” within the context of recovery. Higher levels of identification, often experienced within SIMs, lead to stronger connections characterised by empathy, encouragement, and assistance. The Social Identity Model of Recovery, emphasises the role of social identity in addiction recovery and its impact on individuals’ well-being [43, 44]. This model highlights the importance of identity expression and exploration within recovery spaces. Participants in the current study recognised the significance of connecting with others who share similar identities, such as race, abilities, disabilities, sexualities, and experiences like sex work. The sense of belonging found in SIMs through these shared identifies enhanced the participant’s recovery.

Participant 5’s discussion on the multifaceted nature of their identities underscores the importance of self-expression within recovery spaces. Furthermore, the findings emphasise the value of specialised recovery contexts or SIMs that cater to specific dimensions of identity. These tailored groups offer a platform for individuals to express and explore their identities. This aligns with the idea that recovery is most successful when individuals have strong social support and a sense of identification within their groups, as suggested by the Social Identity Theory [45]. The varied dimensions of identity and sense of belonging discussed in the context of SIMs underscore how diverse identities can transcend societal stigma and contribute to successful recovery journeys [33].

Participants recognised the significance of connecting with others through shared identities. The existence of SIMs catering to various dimensions of identity underscores the value of tailored recovery contexts (Participant 2). These specialised connections helped participants reclaim self-worth, overcome internalised homophobia, and explore their identities (Participants 3 and 8). The participants’ stories highlight that SIMs facilitated a platform for identity expression and exploration, impacting their recovery journey. In contrast, Participant 11’s perspective nuanced the discussion by revealing that being part of a SIM didn’t automatically guarantee a sense of identification with every individual in the group. This insight highlights the complexity of identity dynamics.

#### Meaning in life

SIMs serve as a source of meaning in the lives of SP members. These meetings offer recovery support and serve as recovery resources. The act of finding home groups and sponsors within SIMs adds depth to the recovery journey, which is vital as membership in a homegroup and the role of sponsorship are recognised as ‘active ingredients’ within the effectiveness of 12-Step programs [14]. Participants’ accounts emphasise how SIMs provided meaningful recovery experiences and opportunities for personal growth. The journey from receiving support in SIMs to giving back through service illustrates the cyclical nature of recovery support (Participant 6 and 2). The role of SIMs in helping individuals discover their purpose in supporting others’ recovery journey is evident. Participants found a renewed sense of purpose and meaning by contributing to the meetings that initially helped them (Participants 6 and 2). The participants’ experiences underscore how engagement in SIMs can lead to meaningful engagement in the recovery community for SPs.

#### Empowerment

The empowerment of individuals from SPs within SIMs was evident through their active engagement in creating new spaces that cater to their unique needs and challenges. The data reveals that seven out of 12 participants, who felt marginalised and unsupported in traditional 12-Step meetings, experienced a transformational shift in their sense of agency and empowerment in SIMs. The data highlights that the participants’ commitment to creating innovative and “controversial” (Participant 5) SIMs signifies a profound assertion of their empowerment. In these newly discovered spaces with the 12-Step research landscape, individuals used their agency to shape the recovery environment to their specific needs, and the significance of this empowerment becomes so compelling that it takes precedence over the established ideologies or programs of individual fellowships.

For instance, Participants 10 and 5 collaborated to establish an online SIM called “Black Recovery Matters” for people of colour from various fellowships; they created a designated “safe space” where individuals from marginalised backgrounds could find solace, understanding, and support they did not experience in traditional meetings. These participants took matters into their own hands, defying conventional norms and expectations to establish a platform that prioritises the well-being and recovery of SPs, particularly those facing systemic challenges related to race and ethnicity. This sense of agency and empowerment also extended to forming new fellowships, as discussed by Participant 11, who shared that Crystal Meth Anonymous was created because in other fellowships, they did not feel there was a space to talk openly about their struggles related explicitly to taking meth.

Similarly, after recognising that sex workers were being unfairly judged in traditional meetings, Participant 6’s initiative to assist with creating an online Trans Sex Workers meeting by combining people and literature - which is thought to be controversial in the 12-Step community - from different fellowships showcases empowerment.

They seized the opportunity to challenge the status quo and rectify a critical gap in the support system by creating a space where these marginalised individuals could engage in their recovery journey without fear of judgment or prejudice. These findings contrast with research indicating fellowships can be judgment-free [35].

### Safety

The theme of safety emerged prominently in the analysis, with sub-themes including physical safety, perceived safety, and places of safety. Ten participants highlighted the value of SIMs as safe spaces within the recovery context. They emphasised that SIMs foster a welcoming and secure environment, making it easier for newcomers and likeminded individuals to engage (Participants 5 and 6). Notably, Participant 12 shared a personal experience of feeling unsafe during their recovery journey, underscoring the significance of establishing supportive and safe spaces that empower individuals to settle into their recovery (Participant 12).

Participant 11 introduced a unique perspective on safe spaces, acknowledging the significance of such spaces for certain groups while sharing their viewpoint that such spaces might not be a personal necessity. This perspective prompts a broader discussion on the diverse need for safe spaces in recovery contexts, acknowledging that individual experiences and recovery stages can influence the perceived importance of these spaces (Participant 11). This section of the findings highlights the essential role of safe spaces in facilitating recovery and raises important considerations about their varying relevance across individuals and recovery stages.

### “Special Population Pathways” in 12-Step recovery

In 12-Step recovery, there are various pathways that members from SPs can take. While some prefer traditional meetings, others choose “Special Population Pathways” catering to their needs. This study identified four pathways SPs use to recover: Traditional Pathway, Hybrid Pathway, SIM-Only Pathway, and Outside-Sim Hybrid Pathway.

SP members enjoy exploring all available options and finding the path that best suits their recovery needs. The Traditional Pathway involves attending meetings where individuals primarily identify as addicts or with an issue according to a specific 12-Step fellowship. Individuals may participate in SIMs and traditional meetings in the Hybrid Pathway, as they find support and connection in both settings. The SIM-Only Pathway entails attending SIMs exclusively within a specific fellowship. Lastly, the Outside-SIM Hybrid Pathway combines the support of a SIM formed outside a primary fellowship with traditional fellowship meetings.

Participant 11 followed a Traditional Pathway, connecting more as an addict by attending conventional meetings than focusing on their LGBTQIA+ identity in SIMs. This discovery underscores the way identities can be implemented and manifested within a particular recovery context. This finding suggests that individuals may prioritise different aspects of their identity in their recovery journey, which can impact their choice of support systems. The presence of only two participants among the 12 using a Traditional Pathway demonstrates the diversity in how individuals approach their recovery within the context of 12-step fellowships. It challenges the assumption that a single Traditional Pathway is universally effective and emphasises the need to consider a range of recovery approaches.

Participant 3 adopted a “Hybrid Pathway,” drawing support from LGBTQIA+ SIMs and traditional meetings to address their identities as a father and gay man, highlighting the intricate relationship between one’s identity and their recovery journey. This finding suggests individuals may choose hybrid pathways to meet their unique identity-related needs. The high prevalence of the Hybrid Pathway as the most popular path in the results indicated that a substantial portion of members from a special population preferred a flexible and multifaceted approach to their recovery. This implies that a blended approach which uses various types of meetings in 12-Step fellowships can be effective and offer a comprehensive solution addressing various aspects of the recovery journey.

In contrast, Participant 6 chose a “SIM-Only Pathway,” relying solely on SIMs within their fellowship for recovery. Importantly, the SIM-Only pathway demonstrates that, for some members, 12-Step recovery would not be effective without these meetings. When research discusses the effectiveness of 12-Step fellowships for the recovery of SPs, it is important to investigate and acknowledge the potential role that SIMs contribute to that effectiveness rather than assume it is specific content or processes attributed to a certain fellowship. The “SIM-Only Pathway” within 12-Step fellowships supports the argument that 12-Step effectiveness may not be solely attributed to a fellowship’s specific content or processes but rather its ability to provide free, long-term, easy access, and exposure to recovery-related therapeutic elements [11].

Meanwhile, Participants 2, 5 and 10 pursued an “Outside-SIM Hybrid Pathway,” combining support from a SIM formed outside their main fellowship with their primary fellowship meetings for recovery. Although these novel SIMs, were seen as “controversial” (Participant 5), for using literature from different fellowships, and had its own “sort of jurisdiction” (Participant 10). The existence of these Outside-SIMs further supports the idea that the therapeutic value of these spaces can supersede a 12-Step fellowship’s specific ideology or content. This pathway demonstrates how important it is for individuals to engage with meetings in a way that suits their perceived needs, highlighting the flexibility and adaptability of SIMs as a valuable resource for recovery. These SIMs were created because participants such as black people and transgender sex workers did not feel adequately supported in fellowships.

These diverse Special Population Pathways underscore the complex and multifaceted ways special populations engage in 12-Step recovery. This engagement is influenced by factors such as identity, affiliation, and a profound sense of connection, all of which collectively contribute to tailoring a personalised recovery journey for each individual. Thus, recognising the taxonomy of these distinct pathways within 12-Step recovery is integral to comprehending the intricate dynamics that shape the recovery experiences of special population members. In addition to providing support and community, SIMs play a role in helping individuals explore different recovery pathways beyond fellowships. The typology of these meetings could also be extended to professional settings.

### SIMs and broader experiences of recovery

SIMs could play a significant role in the broader experience of recovery pathways, including specialist treatment. While specialist treatment programs offer a focused and intensive approach to recovery, SIMs could enhance social capital by offering individuals a long-term support network they can engage with throughout their recovery journey. Participant 11 highlighted this point, discussing the value of meeting fellows in SIMs with whom they developed friendships beyond the fellowship. This ongoing support can help individuals maintain their recovery and navigate the challenges they may face [45].

Moreover, SIMs are not isolated from specialist treatment but complement it by providing ongoing support and a sense of community beyond the formal treatment period. Kelly and Yeterian [46] highlight the role of mutual-help groups as adjuncts to specialist treatment, offering additional support and resources to individuals, particularly those with dual diagnoses. For example, Participants 7 and 8 attended SIMs tailored to individuals with specific dual diagnoses or those dealing with addiction in the context of other physical health issues, offering additional support and resources tailored to their needs. SIMs are integral resources, providing support beyond formal treatment.

In addition to providing support and community, SIMs help individuals explore different recovery pathways. Kelly et al. [47] discuss the concept of recovery as an organising paradigm in the addiction field and the need to understand the prevalence and correlates of adopting a recovery identity. SIMs allow individuals to explore different aspects of their identity and find a recovery approach that aligns with their values and needs (Participants 3, 8 and 5). These meetings provide a platform for individuals to discuss and learn about alternative recovery approaches, increasing the likelihood of sustained recovery [7].

SIMs, like general meetings, operate as multifaceted resources. SIMs could contribute to personalised recovery plans and play a role in sustained recovery and relapse prevention. The variety of SIMs in 12-Step meetings could support those in professional treatment to create personalised recovery plans by choosing meetings that resonate with their specific goals. They allow individuals to tailor their recovery journey to their particular challenges and preferences. The current findings highlight that SIMs help people in recovery delve deeper into specific triggers, coping strategies, and life skills, assisting individuals to build a more robust foundation for long-term recovery. Sustained recovery often involves ongoing support [16], which suggests that regular attendance at SIMs can be instrumental in achieving and maintaining sobriety.

## Conclusion

This study highlights the vital role that Special Interest Meetings play within the context of 12-Step fellowships for Special Populations. The challenges these individuals encounter, ranging from stigma to exclusion, have spurred the creation of Special Interest Meetings as spaces that address their distinctive struggles and provide tailored avenues for recovery. The research underscores how Special Interest Meetings foster connections, hope, shared identification, and empowerment among people from Special Populations in recovery. Furthermore, members from Special Populations, who often feel marginalised in traditional 12-Step meetings, have proactively established novel SIMs that cater to their specific needs, promoting inclusivity and safety. The study’s findings also unveil the concept of “Special Population Pathways,” highlighting diverse approaches to recovery. While both traditional and Special Interest Meetings offer supportive elements, Special Interest Meetings provide a unique platform that can significantly enhance the recovery journey for Special Populations. This research emphasises the pivotal role of Special Interest Meetings in enriching recovery experiences for these individuals in professional and non-professional settings. It underscores the importance of acknowledging and addressing their distinct challenges within the broader recovery landscape.

## Limitations

This study is exploratory in nature and limited in scope as it provides insights from a small sample size. While significant associations were observed in this study, it is important to recognise that these findings are not meant to be representative of the wider population of 12-Step members. In conducting this research, I recognise the potential for bias and assumptions due to my dual role as a researcher and 12-Step member. Personal experiences and beliefs could have influenced the findings and interpretation. To address this, transparency was maintained regarding my affiliations, and efforts were made to adopt a reflexive stance. Data triangulation and peer debriefing with a Supervisor was employed to ensure a well-rounded perspective and reduce potential bias. While my insider experience added context, these measures aimed to maintain objectivity and credibility throughout the study.

## Recommendations

Based on the findings presented in this study, further research can explore the effectiveness of Special Interest Meetings and specialist treatment to support Special Populations in their recovery journeys better:

### Explore the dynamics of “tribe” formation in Special Interest Meet**ings**

Future research should investigate the concept of “tribe” formation within SIMs and its impact on the sense of community and relationships among members. This research should also examine the challenges associated with exclusivity, groupthink, and the potential loss of individuality within these specialised meetings. By delving into these dynamics, researchers can provide valuable insights into Special Interest Meetings, including potential drawbacks.

### Research and monitor outcomes

To understand the effectiveness and impact of Special Interest Meetings and specialist treatment for Special Populations, it is recommended to undertake longitudinal studies. These studies should focus on tracking the progress and outcomes of SPs who engage in various recovery approaches like specialist treatment and 12-Step fellowships, or hybrid combinations which include the use of SIMs. Key variables to measure in these longitudinal studies should encompass relapse rates, quality of life improvements, and SPs’ satisfaction levels with their recovery support experiences. This research approach will provide valuable insights into the long-term effectiveness of these interventions and inform ongoing improvements in support for SPs.

### Exploring novel hybrid SIMs in 12-Step recovery

This study identified the emergence of innovative Special Interest Meetings that amalgamate participants and resources from various 12-Step fellowships, representing a novel development in 12-Step recovery practices. Further research is recommended to deepen our understanding of the effectiveness, impact, and potential advantages and hurdles associated with these novel SIMs. This research has the potential to expand the knowledge on recovery strategies and offer insights into how these hybrid SIMs can be optimised and integrated into existing recovery support systems, ultimately enhancing the support available for individuals from special populations.

## Data Availability

The data produced and examined in this study are not accessible to the public to safeguard the anonymity of the participants. However, interested parties can request access to the data from the corresponding author on a reasonable request.
